# Acute Postoperative Unilateral Frontalis Palsy With Spontaneous Resolution After Placement of Mayfield Skull Clamp

**DOI:** 10.5435/JAAOSGlobal-D-20-00123

**Published:** 2020-09-17

**Authors:** Mary Lundgren, Wissam Elfallal, Daniel Park

**Affiliations:** From the Department of Orthopedic Spine Surgery, William Beaumont Hospital (Dr. Lundgren, Dr. Park), and the Department of Neurological Surgery, Oakland University William School of Medicine, William Beaumont Hospital, Royal Oak, MI (Dr. Elfallal).

## Abstract

Cranial holders are used routinely in cranial and spinal surgery with rare reported complications, but frontalis palsy has not been reported as a complication of a Mayfield pin placement. Injury to the temporal nerve, a branch of the facial nerve that supplies the frontalis muscle, is possible because of its subcutaneous nature. A 78-year-old man presented after a fracture dislocation at C7-T1 following a ground level fall. He had progressive axial neck pain and clinical signs of C8 radiculopathy. The patient underwent elective C5-T2 fusion with an open reduction and internal fixation with the use of Mayfield skull immobilization. Postoperatively, he had right unilateral frontalis palsy. The patient was followed clinically for over 12 months and was treated conservatively without surgical intervention or nerve testing. He had spontaneous resolution of palsy with full recovery 2 months postoperatively. Proper placement of the Mayfield skull clamp is key to preventing complications. Knowledge of the landmarks for the temporal nerve assists in safe pin placement to avoid procedural morbidity. Frontalis palsy, if occurs, can be monitored for spontaneous resolution in the postoperative period.

Cranial head holders provide rigid fixation of the calvarium for assistance in instrumented fusion and complex cranial procedures. The Mayfield skull clamp (Integra LifeSciences Corporation, Plainsboro Center, New Jersey) is commonly used. Direct complications from this skull clamp are rare but can incur notable morbidity and mortality without early recognition. Reported complications after skull clamp placement include pin slippage, epidural hematoma,^1,2^ depressed skull fractures,^3^ dural lacerations, air embolism,^4,5^ traumatic cerebral spinal fluid leaks,^6^ wound infections, traumatic superficial temporal artery aneurysm, and middle meningeal arteriovenous fistula.^7^ Demiroz et al^8^ reported an injury to the facial nerve during prone positioning during a spinal procedure that resolved after a course of oral steroids and physical therapy. The authors are not aware of a peripheral facial nerve injury related to Mayfield clamp placement.

The course of cranial nerve VII, the facial nerve, is at risk during procedures because of its subcutaneous course, particularly at the lateral border of the frontalis muscle.^9,10,11,12^ The facial nerve exits the cranium through the stylomastoid foramen, courses through the parotid gland, and divides into the temporozygomatic and cervicofacial divisions and then further into five main branches: temporal, zygomatic, buccal, mandibular, and cervical.^13^ Injury to the temporal branch can result in paralysis and cosmetic deformity of the frontalis, orbicularis oculi, and corrugator supercilii muscles. This case report demonstrates a rare transient, unilateral neurapraxia of the frontalis muscle after Mayfield pin fixation and reviews the landmarks for the identification of the temporal branch of the facial nerve.

## Case Report

**Figure 1:**
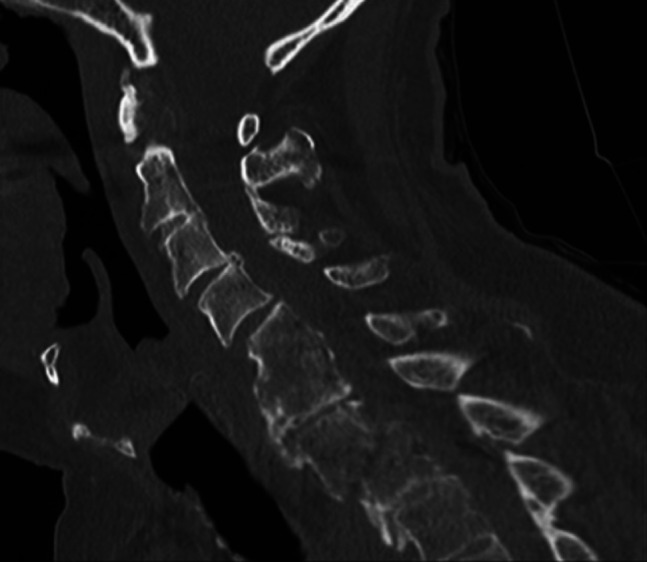
Representative preoperative sagittal CT cut demonstrating an oblique fracture through the C7-T1 ankylosed segment.

**Figure 2:**
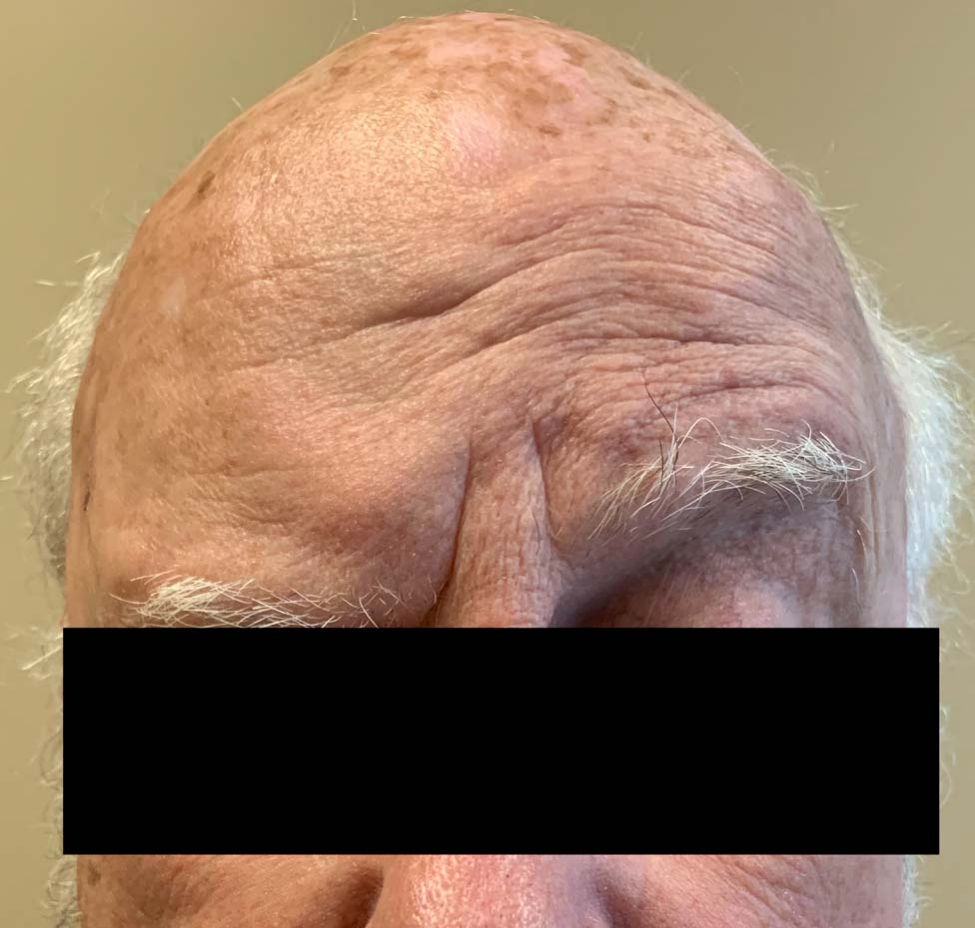
Photograph of the patient attempting to raise bilateral eyebrows. Note the absence of wrinkles or signs of muscle contraction due to right frontalis palsy.

**Figure 3:**
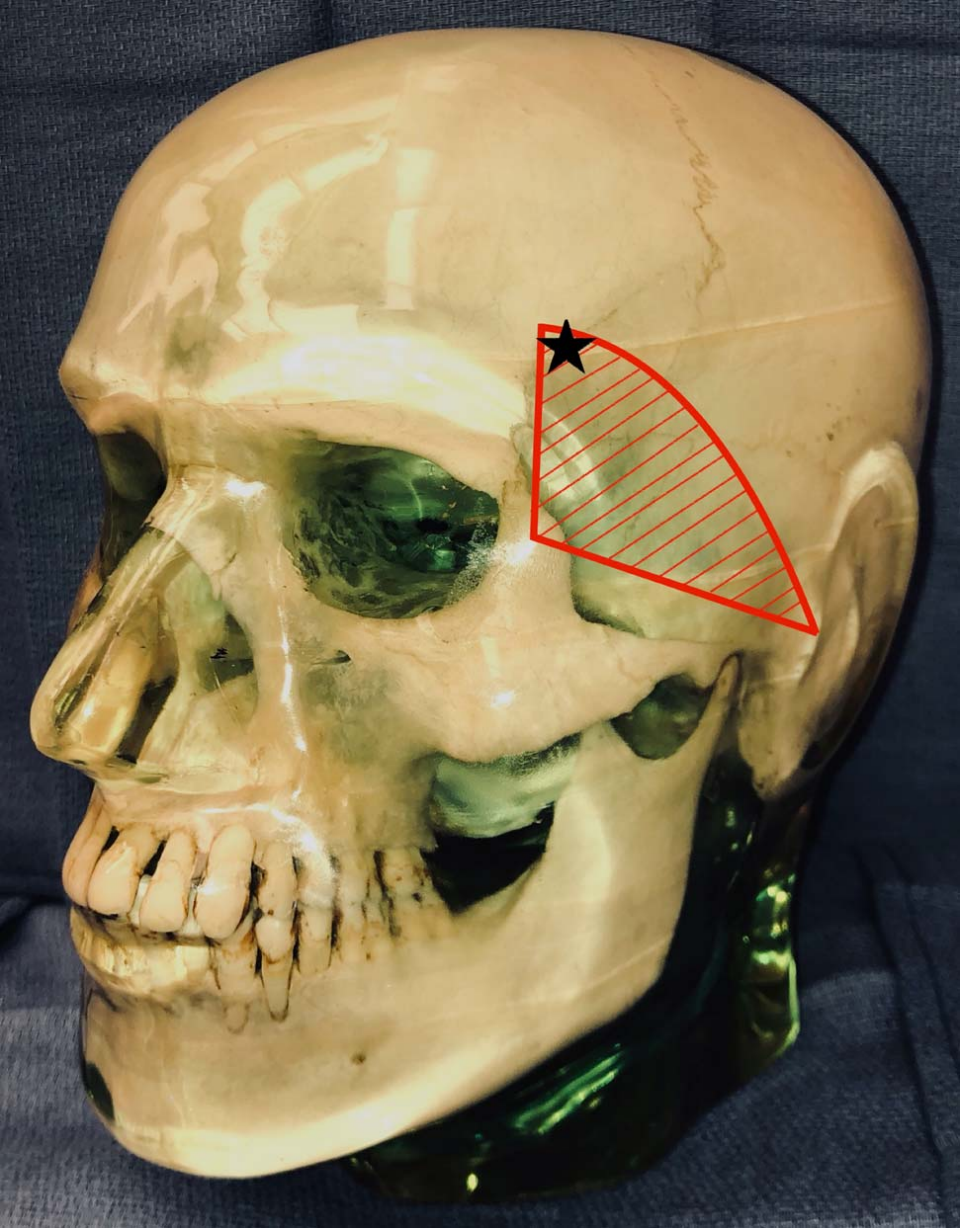
Skull model demonstrating the approximate location of the temporal branch of the facial nerve based on the bony description of Ishikawa^10^ (shaded in red), with a black star demonstrating the approximate location of one of the Mayfield pins in our patient that resulted in frontalis palsy.

**Figure 4:**
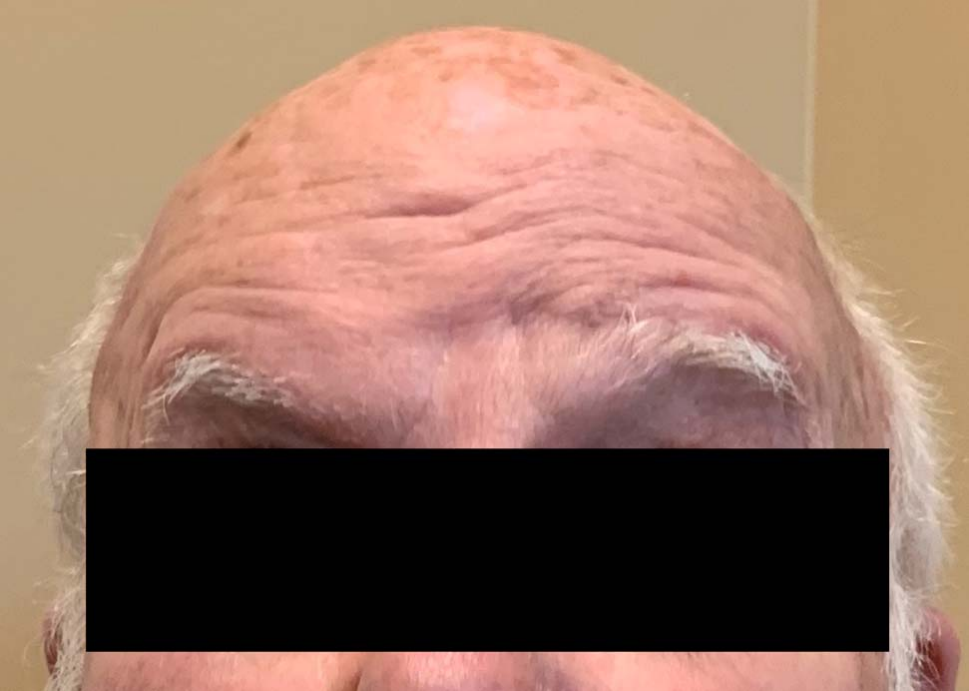
Photograph of the patient raising bilateral eyebrows after 2 months. Resolution of frontalis palsy with wrinkles from frontalis muscle contraction and elevation of the right eyebrow.

**Figure 5:**
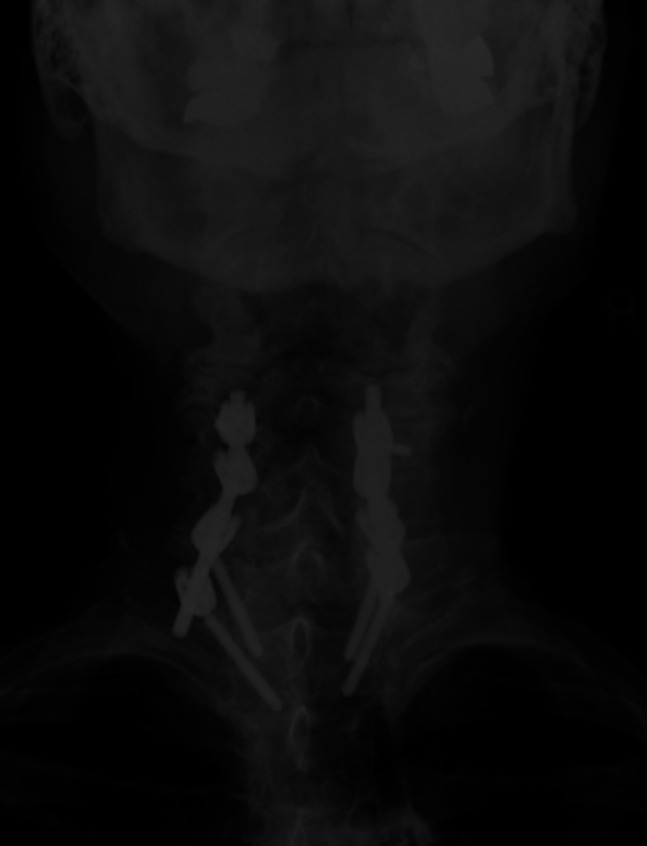
Anterior-posterior radiograph of the patient at 6 months after C5 to T2 posterior cervical fixation.

**Figure 6:**
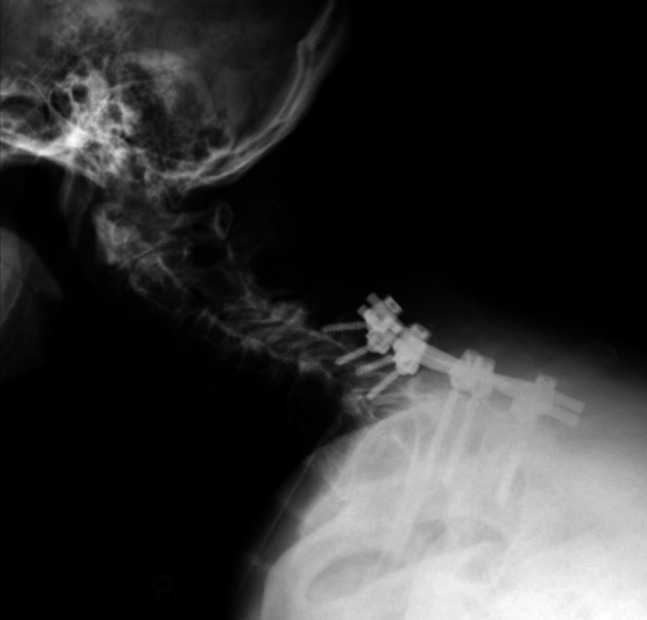
Lateral radiograph of the patient at 6 months after C5 to T2 posterior cervical fixation.

A 78-year-old man with ankylosis presented with a fracture involving the C7-T1 segment (Figure 1) after a ground level fall. Initial symptoms included axial neck pain with C8 radiculopathy and intrinsic muscle weakness. Owing to the fracture and resultant neurologic symptoms, the patient was counseled on cervical thoracic fusion. He underwent a C5 to T2 posterior fusion on an elective basis. Before positioning the patient prone, Mayfield skull clamps were applied. The surgery lasted 122 minutes and was without any intraoperative complications. Postoperatively, it was noted that his right frontalis muscle was unable to contract (Figure 2). The patient's neurapraxia was painless and without signs of infection or injury to the underlying bone. A thorough neurologic examination did not demonstrate any other muscle paresis, including the other muscles innervated by the facial nerve. The pin site was noted to be posterior to the eyebrow (Figure 3). His postoperative course was otherwise uncomplicated. The patient was followed in the outpatient setting, and his frontalis palsy resolved spontaneously without any intervention by 2 months (Figure 4). He healed appropriately with regard to his cervical thoracic fusion (Figures 5 and 6) and had improvement in his clinical symptoms, including improvement in sensation and strength of his hand.

## Discussion

Skull clamp fixation is used to provide stabilization during surgical procedures with limited complications, which can generally be avoided with proper placement. Frontalis palsy has never been reported but underscores the importance of a detailed understanding of facial anatomy, including the at-risk temporal branch of the facial nerve due to its subcutaneous course, particularly at the lateral border of the frontalis muscle.^9,10,11,12^ Using the safe zone of placement illustrated by Beuriat et al^7^ will limit most complications. The safe zone avoids placement of the pin along the course of the facial nerve as it courses through the parotid gland. Pins should be placed along the center line of the calvarium, the region where a sweatband would be worn, with an equal distance between the pins will avoid slippage.

The plastic surgery literature has identified landmarks to estimate the course of the temporal branch of the facial nerve with consideration of anatomical variations, so that injury can be minimized during procedures. Pitanguy and Ramos^9^ described a line starting 0.5 cm inferior to the tragus, the external ear prominence anterior to the concha, connecting to 1.5 cm superior to the lateral eyebrow. Correia et al^11^ described its path along a course from two lines diverging starting at the inferior ear lobe and ending at the lateral eyebrow and to the highest frontal crease. These guidelines are dependent on using the eyebrow as a landmark, which can be variable in the cohort and potentially unreliable. Ishikawa^10^ estimated the course of the temporal branch using bony landmarks. A point 7 cm lateral to the lateral canthus on a line along the zygomatic arch and a point 4 cm superior to the lateral canthus on a line perpendicular to the first line; the temporal branch is estimated to course along a gently curved line connecting these two points.^10^ In our case, the skull clamp was likely placed too anterior and injured the nerve along its course to the frontalis muscle; fortunately, the neurapraxia resolved with time. This was a rare but important complication to recognize to decrease the morbidity and potential cosmetic deformity with the use of skull clamps. Using the projected safe zones described by Beuriat et al^7^ and the general course of the temporal nerve outlined by Ishikawa^10^ should avoid frontalis palsy.

Given the rarity of frontalis palsy, there are no clinical recommendations on the diagnostic and treatment course. Clinical practice guidelines for facial nerve weakness or paralysis, Bell's palsy, could serve as a guideline for diagnostic and treatment options.^14^ These guidelines strongly recommend for oral steroids and against electrodiagnostic testing for incomplete paralysis.^14^ In the event frontalis palsy does occur after placement of the Mayfield skull clamp, the patient can be monitored for improvement in the acute postoperative period with a short course of oral steroids if there are no clinical contraindications.
